# Editorial: Pathology and Oncology Research: addressing publication ethics issues

**DOI:** 10.3389/pore.2024.1611691

**Published:** 2024-02-14

**Authors:** József Tímár, Andrea Ladányi, Anna Sebestyén, László Kopper

**Affiliations:** ^1^ Department of Pathology, Forensic and Insurance Medicine, Semmelweis University, Budapest, Hungary; ^2^ National Institute of Oncology, Budapest, Hungary; ^3^ Department of Pathology and Experimental Cancer Research, Semmelweis University, Budapest, Hungary; ^4^ Arányi Lajos Foundation, Budapest, Hungary

**Keywords:** peer review, publication ethics, scientometry, quality control, transparency

At the Pathology and Oncology Research (POR) journal, Editorials authored by its Editors-in-Chief are a rare publication type. The last time that this journal published such an Editorial was on the 25th anniversary of its launch and indexation in PubMed-Medline [1]. We always felt that a scientific journal must publish research papers and not promotional materials for its own significance. POR was founded and is still owned by the Arányi Lajos Foundation for Modern Pathology, a non-profit Hungarian Foundation established for the support of research in Central and Eastern Europe in the fields of pathology and oncology. Throughout the years, POR has partnered with several well-respected publishers, including Saunders, Springer and recently Frontiers, to support its development and publishing operations. In the past three decades POR has published more than 2,700 articles, has been ranked in Q2 within the SCImago Pathology and Forensic Medicine category since 2000 (67th position in 2022) [2], and received its first Impact Factor (IF) in 2005 (2022 JCR IF: 2.8) [3]. In 2020 POR started operating as a Gold Open Access journal. The journal archive was made fully open access, and the journal has since published 399 articles until December 2023, with an acceptance rate of 20%. The average time to acceptance is 107 days, which is sufficient for rigorous reviewing (it is longer in the case of manuscripts that enter independent review state and much shorter for those that are rejected during prescreening). The low acceptance rate and the suitable decision and acceptance times reflect the dedication of our editors and reviewers in maintaining the high quality and credibility of POR.

Today, publication ethics issues, including, for example, plagiarism or data fabrication, represent a major problem in scientific publishing, and combating them requires enormous efforts from editors and publishers. Furthermore, the appearance of the so-called predatory publishers facilitates the spread of fake scholarly publications. Accordingly, a question arises, what editors, publishers and researchers/authors can do to save the integrity and quality of scientific research and journals. There could be many approaches which require: 1) efforts invested in screening and retracting paper mill/fake papers and uncovering fraudulent review processes by the journals; 2) initiate changes in publication pressure and scientometric-based research funding; and last but not least 3) more transparent and ethical research and publication behavior by the authors.

POR is accredited by and is a member of major publishing regulatory and ethical organizations, adhering to the highest quality standards and best ethical practices. These include COPE (Committee on Publication Ethics) [4] and DOAJ (Directory of Open Access Journals). POR strives to maintain quality in scientific publishing, its Editorial Board hosts well-respected scientists from all around the world. As of December 2023, the Editors-in-Chief work together with 44 Associate Editors and 77 registered Reviewers. Furthermore, using state of the art technology provided by Frontiers, the submissions are screened for editorial quality, such as plagiarism, data fabrication and fraudulent article characteristics, before initiating peer-review. We regularly monitor the submitted as well as the accepted papers. If some of these including already published articles raise a suspicion, we review it thoroughly. In case the authors cannot meet our data request, requirements, we initiate the rejection (retraction in the case of already published papers) of their manuscript/paper. We also try to increase the quality of the journal by launching new Special Issues covering topics of general interest such as the international guidelines for breast cancer diagnosis and treatment [5, 6] or the controversies of liquid biopsies [7, 8], the later guest-edited by the president of the European Society of Pathology. We accept requests only after consultation with the Editors-in-Chief and editorial board members; mainly personal requests for editorial assignments or invited papers. Moreover, POR is constantly increasing its financial support for authors from Central and Eastern Europe and developing countries, having granted until December 2023 the equivalent to USD 99885 in article processing charges discounts since it operates as a Gold Open Access journal.

The quality of POR’s publishing activities since it started operating as a Gold Open Access journal is showcased in the Editors’ Picks collections of POR articles published in 2021 [9] and 2022 [10]. Today it is easy to distribute false information on social media. We, therefore, call the scientific community at large to make their own due diligence on journals and publishers, rather than to rely bluntly on and propagate blacklists published on an anonymous website with hidden agenda, confusing the existing problem of predatory publishing and/or fake/paper mill articles with open access publication.
